# Simultaneous detection and differentiation of *Rice black streaked dwarf virus* (RBSDV) and Southern rice black streaked dwarf virus (SRBSDV) by duplex real time RT-PCR

**DOI:** 10.1186/1743-422X-10-24

**Published:** 2013-01-18

**Authors:** Peng Zhang, Thi Thi Mar, Wenwen Liu, Li Li, Xifeng Wang

**Affiliations:** 1 State Key Laboratory for Biology of Plant Diseases and Insect Pests, Institute of Plant Protection, Chinese Academy of Agricultural Sciences, No. 2, West Yuan Ming Yuan Road, Beijing, 100193, China

**Keywords:** RBSDV, SRBSDV, RT-qPCR, Discrimination, Quantification

## Abstract

**Background:**

The diseases caused by *Rice black streaked dwarf virus* (RBSDV) and Southern rice black streaked dwarf virus (SRBSDV) have been occurring epidemically in China and southeastern Asia in recent years. A sensitive, reliable and quantitative method is required to detect and distinguish for RBSDV and SRBSDV in rice and vector insects.

**Results:**

We developed a sensitive and lineage-specific duplex real time RT-qPCR for detection of RBSDV and SRBSDV in a single or/and double infection in rice samples. The duplex RT-qPCR was optimized using standard samples transcribed by T7 Large Scale RNA Production System *in vitro*. We developed a reliable system for duplex RT-qPCR, in which its co-efficiency of RBSDV and SRBSDV, were 91.6% and 90.7%, respectively. The coefficient of determination was more than 0.990; the slope of linear equation was −3.542, and −3.567, respectively. Out of 30 samples collected in North and Central China, which were suspected to be infected with these two viruses, 10 samples were detected RBSDV positive by RT-PCR and 12 samples by RT-qPCR. No mixed infections were found. Simultaneously, out of total 60 samples collected from Southern China, which were also suspected to be infected with these two viruses, 41 samples were determined SRBSDV positive by RT-PCR and 47 samples by RT-qPCR. Also in this case no mixed infections were found. The rice genes eEF-1a and UBQ5 were selected as internal controls for quantification assay also performed as good expression stability.

**Conclusion:**

The duplex RT-qPCR assay provided as a sufficiently sensitive, specific, accurate, reproducible and rapid tool for the detection and differentiation of RBSDV and SRBSDV. The RT-qPCR assay can be used in routine diagnostic of these two viruses in order to study the disease epidemiology in rice crops.

## Background

In major rice-growing countries, rice viral diseases have been occurring one after another and have inflicted widespread damage over huge areas. More than 15 rice viruses, especially planthopper or leafhopper transmitted viruses have reached epidemic proportions in many countries and have caused serious damage in rice [1]. In China, major outbreak of *Rice black streaked dwarf virus* (RBSDV) was recorded after 1963, when double-cropping of rice became common in Southern China and total production started to greatly increase [2,3]. A disease with similar symptoms of RBSDV was first observed in Guangdong Province, Southern China in 2001. The causal agent of the disease was identified as Southern rice black-streaked dwarf virus (SRBSDV), a tentative species in the genus *Fijivirus*, family *Reoviridae*[4]. SRBSDV has recently spread throughout Southern China and Vietnam [5,6]. The outbreak of SRBSDV in 2009 and 2010 caused serious yield losses in the Southern and Central region of China and in the Northern parts of Vietnam. Nearly 12 million hectares of domestic paddy fields were affected in 2010 in China [7]. Currently, SRBSDV is also known to occur in a number of countries in Southeastern Asia, including Vietnam and Thailand [7,8].

RBSDV occurs mainly in China, Japan and Korea [1]. It is a member of the genus *Fijivirus* within the family *Reoviridae*[9,10]. The virus is transmitted naturally to rice, maize, barley and wheat in a persistent propagative man-ner by three different species of planthopper, *Laodelphax striatellus*, *Unkanodes sapporona* and *U. albifascia*[11-13]. On the other hand, SRBSDV can be transmitted efficiently by the white-backed planthopper (*Sogatella furcifera*, Hemiptera: Delphacidae), which is not the main vector of RBSDV. Beside rice, maize (*Zea mays*), barnyard grass (*Echinochloa crusgalli*), flaccid grass (*Pennisetum flaccidum*) and *Juncellus serotinus* are also the hosts of SRBSDV [6,14].

Both RBSDV and SRBSDV have similar genome structure containing with 10 segments of dsRNA that encoded at least six putative structural proteins (P1, P2, P3, P4, P8, and P10) and five putative nonstructural proteins (P6, P7-1, P7-2, P9-1 and P9-2) [4,6,10,15]. Rice plants infected by either RBSDV or SRBSDV show typical stunting, dark green leaves and small enations on stem and leaf backs [7,12]. However, sometimes these two viruses can be confused with each other and the disease is also very difficult to distinguish with the diagnostic based on symptoms, because the symptoms of these two diseases may vary according to the infection of different growth stages.

Some studies have reported successful methods of RBSDV and SRBSDV detection, including reverse transcription-polymerase chain reaction (RT-PCR), reverse transcription loop-mediated isothermal amplification (RT-LAMP), indirect enzyme-linked immunosorbent assay (ELISA), and indirect dot-immunobinding assay (DIBA) [16-19]. Because information on the virus content in local fields is very important for forecasting and releasing warning schemes to advise farmers on the potential threat to their crops, a sensitive, reliable and quantitative method is required to detect and distinguish RBSDV and SRBSDV in rice and vector insects.

Nowadays, real-time PCR technology has proved an efficient tool for quantitative detection of many plant RNA and DNA viruses [20-25]. Here, we reported that the quantitative RT- qPCR method able to reliably discriminate and quantify RBSDV and SRBSDV in rice. This presented method will provide better possibilities for monitoring the virus numbers and contents in local fields and estimating the extent of primary infections, and then forecasting the outbreaks of these two rice viral diseases.

## Results

### Standard curves of RT-qPCR

**Figure 1:**
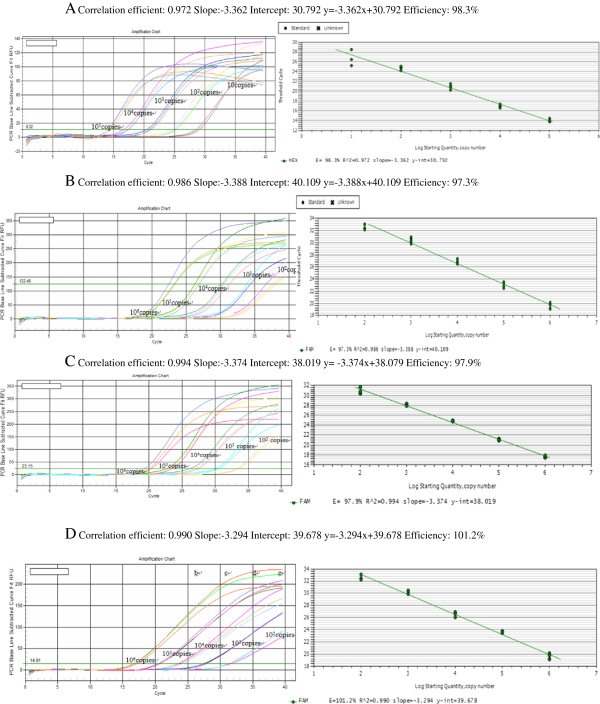
**Standard curve for TaqMan real time RT-PCR amplification of standard ssRNA (viral transcripts).** (**A**) Amplification plots showing the testing in triplicate of a 10-fold dilution series containing standard ssRNA of RBSDV at: 1x10^5^(**a**); 1x10^4^(**b**); 1x10^3^ (**c**); 1x10^2^(**d**); 1x10^1^(**e**) template copies per reaction. (**B**) Amplification plots showing the testing in triplicate of a 10-fold dilution series containing standard ssRNA of SRBSDV at: 1x10^6^(**a**); 1x10^5^(**b**); 1x10_4_ (**c**); 1x10^3^(**d**); 1x10^2^(**e**) template copies per reaction. (**C**) Amplification plots showing the testing in triplicate of a 10-fold dilution series containing standard ssRNA of eEF-1a at: 1x10^6^(**a**); 1x10^5^(**b**); 1x10^4^ (**c**); 1x10^3^(**d**); 1x10^2^(**e**) template copies per reaction. (**D**) Amplification plots showing the testing in triplicate of a 10-fold dilution series containing standard ssRNA of UBQ5 at: 1x10^6^(**a**); 1x10^5^(**b**); 1x10^4^ (**c**); 1x10^3^(**d**); 1x10^2^(**e**) template copies per reaction.

The linear range of quantification of the one-step RT-qPCR assay for RBSDV was determined by using ten-fold serial dilutions of the standard ssRNA ranging from 10 to 1 × 10^5^ copies to determine the end-point limit of detection and the linearity of the assay (Figure 1A). Ct-values were measured in triplicate and plotted against the known copy numbers of the standard sample. The standard curve covered a linear range of five orders of magnitude. The slope (−3.362) and the correlation coefficient (*R*^2^ = 0.972) of the standard curve showed that this assay could be used to quantify target RNA in infected rice tissues. Dilution curves were obtained with total RNA from RBSDV infected rice and their amplification efficiency was 98.3%.

A standard curve of quantification of SRBSDV, eEF-1a, and UBQ5 gene in rice were developed respectively as described above (Figure 1B, C, and D). According to the differences of original standard sample concentration, we used different copy ranges to determine the standard curves as follow: The linear ranges of SRBSDV, eEF-1a, UBQ5 were 10^6^ – 10^2^ copies, 10^6^ – 10^2^ copies, 10^6^ – 10^2^ copies respectively; the slope and correlation coefficient were −3.388, 0.986; -3.374, 0.994; -3.294 0.990, respectively.

### Optimization of duplex RT-qPCR

**Figure 2:**
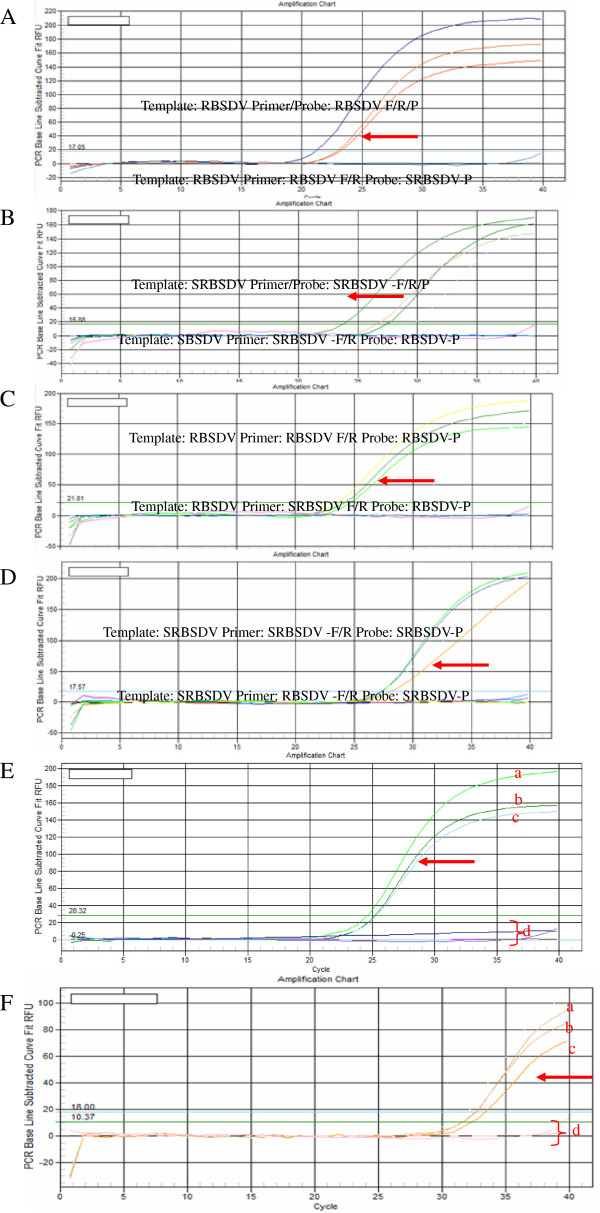
**Optimization of duplex RT-qPCR The concentration of template of RBSDV or SRBSDV were 105 copies.** The arrow indicated positive signal (P), otherwise, negative single(N). **A**: Specific for RBSDV-P probe. P:Standard sample of RBSDV as template; RBSDV-F/R/P as primer and probe. N: Standard sample of RBSDV or H2O as template; RBSDV-F/R as primer; SRBSDV-P as probe. **B**: Specific for SRBSDV-P probe. P:Standard sample of SRBSDV as template; SRBSDV-F/R/P as primer and probe. N: Standard sample of SRBSDV or H2O as template; SRBSDV-F/R as primer; RBSDV-P as probe. **C**: Specific for RBSDV-F/R primer. P:Standard sample of RBSDV as template; RBSDV-F/R as primer and RBSDV-P as probe. N: Standard sample of RBSDV or H2O as template; SRBSDV-F/R as primer; RBSDV-P as probe. **D**: Specific for SRBSDV-F/R primer. P:Standard sample of SRBSDV as template; SRBSDV-F/R as primer and SRBSDV-P as probe. N: Standard sample of SRBSDV or H2O as template; RBSDV-F/R as primer; SRBSDV-P as probe. **E**, **F**: Optimization concentration of primer and probe (**E**) P: standard sample of RBSDV as template. concentration of upstream, downstream primer and probe at **a**)200 nM 200 nM 400 nM; **b**) 200 nM 200 nM 200 nM; **c**) 100 nM 100 nM 200 nM. No positive signal (**d**): H2O as template. (**F**) positive: standard sample of SRBSDV as template. concentration of upstream, downstream primer and probe at **a**) 200 nM 200 nM 400 nM; **b**) 100 nM 100 nM 200 nM; **c**) 200 nM 200 nM 200 nM. No positive signal (**d**): H2O as template.

We have developed the assays with different concentration of specific primers and probes. The primers and probes were absolutely specific for each particular virus. No cross specificity was recorded neither in case of RBSDV nor SRBSDV (Figure 2-A, B, C, D) and proper concentration of primers and probes were also determined. Based on the intensity of detective signal, we got a good setup for the duplex RT-qPCR: 0.4 μl (200 nM) for each of RBSDV-F/R and SRBSDV-F/R, and 0.4 μl (200 nM) and 0.8 ul (400nM) for RBSDV-P, SRBSDV-P, respectively. This step was run at 42°C for 5 min. PCR was performed with the hot-start *Taq* polymerase included an enzyme activation step (95°C for 5 s) followed by 40 cycles of denaturation/annealing–extension (10 s at 95°C; 30 s at 60°C).

### Standard curves of duplex RT-qPCR

**Figure 3:**
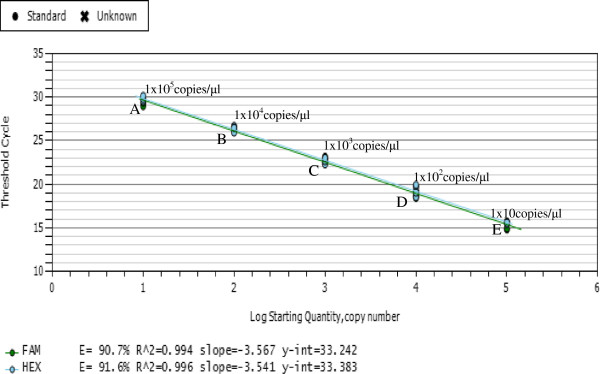
**Detection limits and amplification efficiency of duplex RT-qPCR.** Detection limits and amplification efficiency of the multiplex RT-qPCR for detection of RBSDV and SRBSDV. The standard curves were established by five gradient, in which every gradient contained 3 replicates in duplicate of a 10-fold dilution series containing mixed standard ssRNA of RBSDV and SRBSDV at: 1x10^5^ (**A**); 1x10^4^ (**B**); 1x10^3^ (**C**); 1x10^2^ (**D**); 1x10(**E**) template copies per reaction.

The linear range of quantification of the one-step dup-lex RT-qPCR assay for segment RNA4 of RBSDV and SRBSDV were determined by using ten-fold serial dilutions of the mixed standard ssRNA as template ranging from 10 to 1 × 10^5^ copies to determine the end-point limit of detection and the linearity of the assay (Figure 3). The program was setup for viral RNA at 42°C for 5 min. PCR was performed with the hot-start Taq polymerase included an enzyme activation step (95°C for 5 s) followed by 40 cycles of denaturation, annealing and extension (10 s at 95°C; 30 s at 60°C). Ct-values were measured in triplicates and plotted against the known copy numbers of the standard sample. The standard curve covered a linear range of five orders of magnitude. The slope and the correlation coefficient of the standard curve of duplex RT-qPCR showed that this assay could be used to quan-tify and distinguish target mixed RNA of RBSDV and SRBSDV in infected rice tissues. Dilution curves were obtained with total RNA from infected rice and their amplification efficiencies were 91.4% and 90.7%, respectively. This RT-qPCR assay enabled detection of as few as 10 gene copies in mixed RNA standard samples.

### Detection and relative quantification of RBSDV or SRBSDV in rice samples

Total RNA of field rice samples treated with DNase I, was used for detection by one-step duplex RT-qPCR and RT-PCR [26]. Table 1 shows the results of using duplex RT-qPCR and RT-PCR to check the possible infected samples by only one virus or by the two viruses simultaneously. Among the samples infected by RBSDV from Northern and Central China (Shandong Province and Henan Province), we got the same results with RT-PCR, as no mixed infections were found. Among the samples infected by SRBSDV from Southern China (Hubei Province, Jiangxi Province, Hunan Province, and Yunnan Province), we also got the same results with RT-PCR, as no mixed infection was found either. Simultaneously, we found that RT-qPCR is more sensitive in comparison to RT-PCR.

**Table 1:** Detection of RBSDV and SRBSDV from rice samples collected from six provinces of China by RT-PCR and RT-qPCR

	**Collection Sites**	**Number of samples**	**Duplex real-time RT-PCR positive**		**Duplex RT-PCR positive**	
			**Sole infection**	**Mixed infection**	**Sole infection**	**Mixed infection**
North and central China	Henan	15	RBSDV/12	0	RBSDV/10	0
	Shandong	15	RBSDV/15	0	RBSDV/12	0
Southern China	Hubei	15	SRBSDV/15	0	SRBSDV/15	0
	Hunan	15	SRBSDV/6	0	SRBSDV/4	0
	Jiangxi	15	SRBSDV/14	0	SRBSDV/13	0
	Yunnan	15	SRBSDV/12	0	SRBSDV/9	0
	Total	90	73	0	63	0

In this study, the minimum detection limit of this duplex RT-qPCR assay was 10 copies. We collected a total of 90 field rice samples showing dwarf symptoms from 6 provinces of China in 2012, 22 and 41 samples were detected as RBSDV infected by RT-PCR and RT-qPCR respectively. RBSDV was mainly detected in Northern and Central China (Shandong and Henan province). On the other hand, SRBSDV was detected in the samples collected from Southern China. Forty one and 47 samples were detected as SRBSDV infected by RT-PCR and RT-qPCR, respectively. So, this duplex RT-qPCR assay was able to detect more positive samples than RT-PCR.

**Figure 4:**
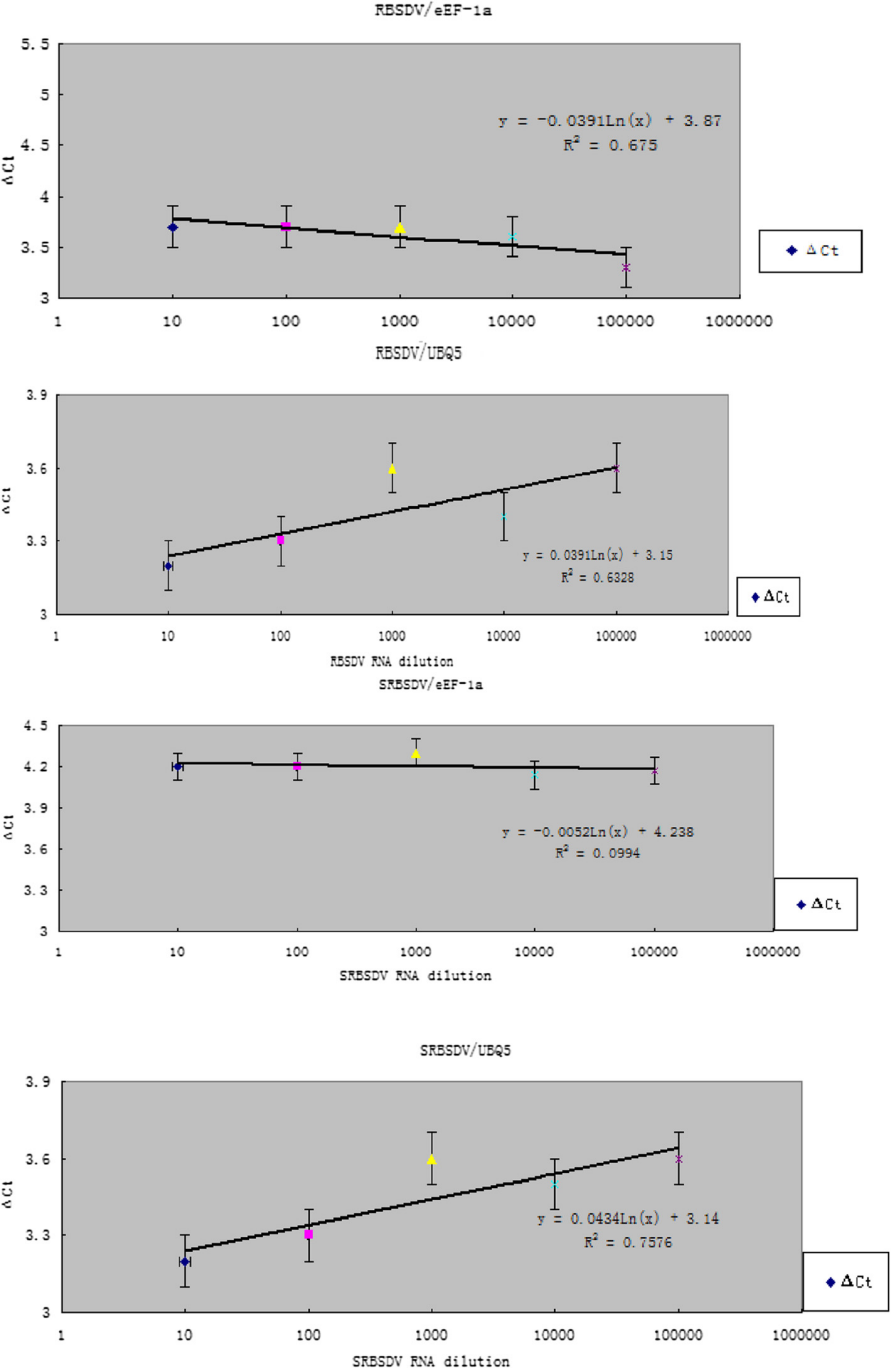
**The linear correlation of dilution factor with the ΔCt value. A**: linear equation of dilution factor of RBSDV RNA with the ΔCt value of RBSDV to eEF-1a, the slope is -0.0391; **B**: linear equation of dilution factor of RBSDV RNA with the ΔCt value of RBSDV to UBQ5, the slope is 0.0391; **C**: linear equation of dilution factor of SRBSDV RNA with the ΔCt value of SRBSDV to eEF-1a, the slope is -0.0052; **D**: linear equation of dilution factor of SRBSDV RNA with the ΔCt value of SRBSDV to UBQ5, the slope is 0.0434.

Then, we compared the PCR efficiency of RBSDV or SRBSDV to both of internal references, using RNA extracted from samples infected by RBSDV or SRBSDV based on the results of RT-qPCR. We found that the dilution factor of RBSDV RNA had a linear correlation with the ΔCt value of RBSDV to eEF-1a or UBQ5 and the slope were −0.0391 and 0.0391 respectively, near to 0, confirming that there were consistencies of RT-qPCR efficiency between RBSDV and eEF-1a or UBQ5 (Figure 4A and (B). Meanwhile, we did a same assay on SRBSDV, and got similar results (Figure 4C and (D): the slope was −0.0052, 0.0434 respectively, also near to 0, confirming that there were consistencies of RT-qPCR efficiency between SRBSDV and UBQ5 or eEF-1a [27,28].

**Figure 5:**
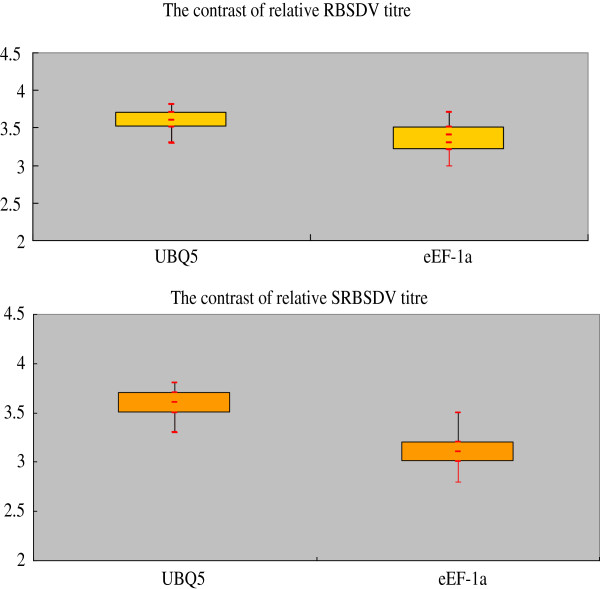
The relative RBSDV and SRBSDV titer levels in the positive field samples determined by relative quantification using UBQ5 and eEF-1a.

Besides being extremely powerful technique, RT-qPCR suffers from certain pitfalls, to solve this problem, the normalization with a reference or housekeeping gene (s) is the most important. The expression of reference gene used for normalization in RT-qPCR analysis should remain constant between the cells of different tissues and under different experimental conditions; otherwise, it can lead to erroneous results. So we used the date of positive samples to check the stability of UBQ5 and eEF-1a. The virus titer was calculated under relative quantification with the rice UBQ5 and eEF-1a genes. To RBSDV and SRBSDV, the trends of the two reference genes reflecting the deviation of date were basically same (Figure 5). It was indicating that the UBQ5 and eEF-1a were suitable to use as reference genes in the assay of RT-qPCR, which is in agreement with previous reports [24,28]. Hence, it further confirms the validation for duplex RT-qPCR set up in this study. Also these results support the necessity of the correct choice of reference genes for valid experimental data as reported elsewhere [28,29].

## Discussion

The rice diseases caused by RBSDV and SRBSDV have been responsible for significant economic loss in recent years in China and other Asian countries. Diseases caused by RBSDV and SRBSDV were latent and difficult to diagnose at an early stage, but very destructive at a late stage. Therefore, these diseases need to be monitored and diagnosed at their early stages for effective mitigation of loss and risk assessment of infected rice paddy field [5,6,8]. RT-PCR, RT-LAMP, and serological methods are most commonly used for detecting plant viruses, but by none of these methods it is possible to determine a virus contents in plant and insects vector which are key factor for disease epidemics [17-19,24]. In this study, we established a duplex RT-qPCR assay for the detection, discrimination, and quantification of RBSDV and SRBSDV in rice plants. Using two different fluorescence signals, this method could detect quantitatively RBSDV and SRBSDV in a one reaction tube at the same time. Comparing with RT-PCR, this assay showed higher specificity and sensitivity, and could be a useful method for epidemiological studing and forecasting and controlling the diseases (Table 1).

In RT-qPCR used in discrimination and quantification of several viruses [20,24,29], the Ct value is a parameter reflecting the quantity of template present in the reaction. Usually, lower Ct values indicate a high concentration of template and higher Ct values indicate a low concentration of template [30]. This study showed that standard serial dilution curves were obtained with high RT-qPCR efficiency for RBSDV and SRBSDV (98.3 and 97.3%, respectively). The efficiency proved the balance among enzyme, dNTPs, primers and templates. In the duplex RT-qPCR, we got high co-efficiency of SRBSDV and RBSDV, which were 90.7% and 91.6%, respectively. The rationality of linear equation was determined by the coefficient of determination (R^2). Usually, R^2 value needs to be between 0 and 1, higher value indicates its higher rationality. In this study, the R^2 values of RBSDV and SRBSDV were 0.994 and 0.996 respectively. We therefore suggested that this duplex RT-qPCR could be used to detect quantitatively RBSDV and SRBSDV in routine virus diagnosis.

The rice UBQ5 and eEF-1a genes were selected as internal controls for quantification assay. UBQ5 and eEF-1a were the most stable across all the tissues and all growth stages examined as reference genes [28]. RNA extraction errors, template loading deviations, and variations of reverse transcription efficiency could be eliminated from data analysis by quantification of the UBQ5 and eEF-1a genes. Simultaneously, the relative contents of RBSDV and/or SRBSDV in infected leaves of field samples could be determined by relative quantification.

Our results showed that the distribution of RBSDV and SRBSDV induced diseases were different in China. RBSDV induced disease was found in North and Central China (Shandong and Henan province), but SRBSDV mainly in Southern China (Hubei, Jiangxi, Hunan and Yunnan province). One of the reasons might be related to the biological characteristics of their vector insects [31]. RBSDV is transmitted efficiently by small brown planthopper (*L. Striatellus*), which is believed to show strong indigenousness, overwinter as nymphs or eggs in most rice grown areas; feed on wheat plants in the spring, and migrate to rice seedling after wheat harvest [32], while SRBSDV can be transmitted exclusively by white backed planthopper (*S. furcifera*) [5], a typical immigration pest not overwintering in most regions of China [33,34]. The Southern China is on its immigration path and one of its overwintering regions [33,35]. The abundances of this planthopper may cause break out of SRBSDV in Southern China and/or other Southeastern Asia countries. Although white-backed planthopper can migrate to Central and Northern China on the early or mid-August, rice plants reach a booting stage with higher resistance to virus infection.

## Conclusions

Rice diseases induced by RBSDV and SRBSDV have caused a great loss in the South China and Southeastern Asia. A sensitive and quantitative method has been established to detect and distinguish two viruses in rice. Compared to RT-PCR, RT-qPCR is more sensitive and reliable. We proved that the specificity of primers/probes in the duplex RT-qPCR assay can effectively resolve the problem of mismatches and avoid false negatives and distinguish the RBSDV and SRBSDV. Using this technology, we could detect the possibly infected samples by only one virus or the two viruses at the same time. To the point, one requisite of RT-qPCR is to normalize the data with internal reference genes that is invariant regardless of treatment, such as virus infection, which takes into an account the potential error of RNA extract, template loading, and variation of reverse transcription efficiency. From the assay, we got that UBQ5 and eEF-1a were stable in this experiment. This study clearly demonstrated the potential usefulness of TaqMan probe based RT-qPCR assay for detecting and distinguishing of RBSDV and SRBSDV.

## Materials and methods

### Plant material and virus sources

Rice plants infected with RBSDV or SRBSDV were collected from Jiangsu and Guangzhou provinces of China in the growing seasons of 2008 and 2010 respectively. All the samples had been tested previously by RT-PCR [16], and stored at −70°C. Ninety field rice samples showing stunting, darkening of leaves, and waxy white galls along the veins on the underside of leaf blades and surface of sheaths were collected from Henan, Shandong, Hubei, Hunan, Jiangxi and Yunnan provinces of China in 2012, and stored in a freezer at −70°C.

### RNA extraction

Total RNA were extracted using the RNAiso Reagent (TaKaRa Dlian, China) as the protocol: grind the 0.1 g of leaf with the liquid nitrogen in the mortar, add 1 ml Trizol reagent to the power, put them at the normal temperature for 5 min; transform the liquid to the 1.5 ml tube, add 200 μl chloroform was added, the tubes were shaken for 15 s, lay aside 15 min at −20°C; centrifuged at 12000 rpm for 15 min; the supernatant was transferred into a new tube, equal isopropanol to supernatant was added and the samples were left at normal temperature 30 min; centrifuged 12000 rpm for 15 min; the precipitate was washed using 1 ml of 75% ethanol; the ethanol was removed at Rapid Glass Dryer; 40 μl DEPC H2O were added and the samples were stored at −70°C.

Only of the most critical points in the RT-qPCR experiment is to use only RNA of high purity and high integrity. The concentration of RNA was measured by NanaDrop-1000 (NanoDrop Technologies, USA). The 260/280 ratio should be between 1.9-2.1, and the 260/230 ratio should be greater than 2.0.

### Primers and probes design

**Figure 6:**
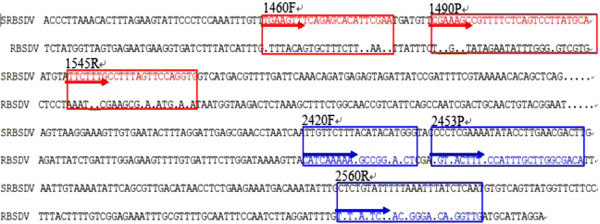
**Localization of primers and probes.** Primers and probes were selected with specificity for the positive-sense strand of RNA4 of RBSDV and SRBSDV. The blue script indicated the primer and probe of RBSDV, and Red script indicated the primer and probe of SRBSDV. Arrow directing indicated the start position from 5’ to 3’ end.

Primers and TaqMan probes were designed to develop a RT-qPCR detection system according to specific conditions, such as high melting temperature (*Tm*) and a shorter amplification production. Alignment of positive-sense strand of RNA4 of RBSDV and SRBSDV was performed with Vector NTI, primers and probes were then designed in silico using Primers Express software (Applied Biosystems, USA). The resulting primers and probes are shown (Figure 6 and Additional file 1: Table S1 and Additional file 1: Table S2) as follow:

To the RBSDV, the first pair of primers of RBSDV: RBSDV 4-F: 5’- GCA AAC GCT CGT CAT CTA AG -3’ (upstream *Tm* = 56°C) and RBSDV 4-R: 5’-CCA CCA AAC GCT ATT TCA CT-3’ (downstream *Tm* = 56°C) correspond to the positive-sense strand of RNA4 of RBSDV (GenBank accession number AJ409146.1), and were expected to amplify a fragment of 903 bp for the standard sample. For fluorescence detection, one primer–probe combination was also selected from combinations proposed by the PRIMER EXPRESS software (Applied Biosystems, USA) according to the manufacturer’s instructions. The first primer–probe combination was designed: 2420 F:5’-CAT CAA AAA AGC CGG AAG CT-3’ (upstream Tm = 57°C), 2453 T:5’-CGT CAC TTT ACC ATT TGC TTG GCG ACA-3’ (TaqMan probe Tm = 70°C), and 2560R:5’-CAA CCA TGA TCC CTG TAA GAA TAA AA -3’ (downstream *Tm* = 57°C) targeting the conserved region within the positive-sense strand of RNA4 of RBSDV (GenBank accession number **AJ409146.1**). RBSDV probe was labeled at the 5’end with exachlorofluorescein (HEX, excitation wavelength 535 nm, emission wavelength 556 nm) and Black Hole Dark Quencher 1 (BHQ-1) at the 3’ end. The first pair of primers for SRBSDV: SRBSDV 4-F: 5’- ACG CTG ATA CCA ACA GAC CA -3’ (upstream *Tm* = 56°C) and SRBSDV 4-R: 5’- TTT AGC ACC AAG AAA GAC GA -3’ (downstream *Tm* = 54°C) correspond to the positive-sense strand of RNA4 of SRBSDV (GenBank accession number FN563992.1), and were expected to amplify a fragment of 1171 bp for the standard sample. For fluorescence detection, one primer–probe combination was selected from combinations proposed by the PRIMER EXPRESS software (Applied Biosystems, USA) according to the manufacturer’s instructions. The first primer–probe combination was designed: 1460 F: 5’- TGA AGT TTC AGA GCA CAT TCG AA -3’ (upstream *Tm* = 55°C), 1490 T: 5’- CGA AAG CCG TTT TCT CAG TCC TTA TGC A -3’ (TaqMan probe *Tm* = 70°C), and 1545R: 5’- CAC CTG GAA CTA AAG GCA AAG AA -3’ (downstream *Tm* = 61°C) targeting the conserved region within the positive-sense strand of RNA4 of SRBSDV (GenBank accession number **FN563992.1**). Primers and probes were purchased from Invitrogen Co. Ltd. SRBSDV probe were labeled at the 5’end with 6-carboxy-fluorescein (FAM; excitation wavelength 494 nm, emission wavelength 521 nm) and Black Hole Dark Quencher 1 (BHQ-1) at the 3’ end, respectively.

Simultaneously, we chose the Ubiquitin 5 and eEF-1a as the rice housekeeping genes, because of the highest stability across all the tissue samples examined [28]. The pair of primers for rice ubiquitin 5 gene (GenBank accession number **AK061988**) is designed as follow: UBQ-F: 5’-CTC GCC GAC TAC AAC ATC C-3’ (upstream *Tm* = 56°C) and UBQ-R: 5’-AGG GCA TCA CAA TCT TCA CA-3’ (downstream *Tm* = 49°C) corresponding to nucleotides of the rice ubiquitin 5 gene [24], and were expected to amplify a fragment of 460 bp for the standard sample. The primer–probe combination: 533 F: 5’-AGT GCG GCC TCA CCT ACG T- 3’ (upstream *Tm* = 63°C), 557 T: 5’-ACC AGC AGG CTT AGG CGT AGG CT-3’ (TaqMan probe *Tm* = 75°C) and 581R: 5’-CCG CCC CCA AAG AAC AG-3’ (downstream *Tm* = 58°C) was designed to target the rice ubiquitin 5 gene as an internal control. TaqMan probe was labeled with 6-carboxyfluorescein (FAM, excitation wavelength 494 nm, emission wavelength 521 nm) at the 5’ end and Black Hole Dark Quencher 1 (BHQ-1) at the 3’ end. The pair of primers for the rice eEF-1a gene (GenBank accession number **AK061464.1**) was shown as follow: eEF-1a-F: 5’- GAC AAG ATT CCC TTC GTT CC -3’ (upstream *Tm* = 54°C) and eEF-1a-R: 5’- TGT AAA TAC CCG CAT TCC AC -3’ (downstream *Tm* = 55°C) corresponding to the sequence of the rice eEF-1a gene, and were expected to amplify a fragment of 945 bp for the standard sample. The primer–probe combination: 772 F: 5’- CCC AAG AGG CCA TCA GAC A - 3’ (upstream *Tm* = 53°C), 793 T: 5’- CCC CTG CGT CTT CCC CTT CAG G -3’ (TaqMan probe *Tm* = 69°C) and 840R: 5’- GCC AAT ACC ACC GAT CTT GTA CA -3’ (downstream *Tm* = 61°C) was designed to target the rice eEF-1a gene as another internal control. TaqMan probe was labeled with 6-carboxyfluorescein (FAM, excitation wavelength 494 nm, emission wavelength 521 nm) at the 5’ end and Black Hole Dark Quencher 1 (BHQ-1) at the 3’ end.

### Preparation for viral RNA standards of one-step RT-qPCR

RNA transcripts were synthesized *in vitro*, inserting the cDNA fragments of RBSDV, SRBSDV, eEF-1a and UBQ-5 into the pGEM T-EASY (Promega, USA) respectively, and then transforming them into competent cell of trans 5a (Trans, China), The right inserted PCR products was monitored by gel electrophoresis of restriction enzyme cleavage.

Positive single strand RNA was transcribed using the RiboMAX Large Scale RNA Production Systems-T7 Kit (Promega, USA), using 2 μg linearzed plasmid DNA as template, then treated by DNaseI at 37°C for 20 min, purifying the RNA by RNAclean kit (BioTeke,China). The purified RNA was quantified using NanoDrop ND-1000.

### One-step RT-qPCR assay and optimization for RBSDV, SRBSDV, UBQ5 and eEF-1a

One-step RT-qPCR reactions were performed in a final volume of 20 μl using the One-step PrimeScript RT-PCR Kit (TaKaRa Biotech., China) according to the manufacturer’s instructions. The reactions carried out with 2 μl of total RNA were performed on the Bio-Rad iCycler IQ Real-Time PCR Detection System. During the amplification process, the fluorescence intensity of the reporter dye (FAM or HEX) was recorded. These data allowed calculation of the normalized reporter signal, which was linked to the amount of product amplified. The threshold cycle (Ct-values, number of cycles for the fluorescence to reach the threshold) referred to the number of amplification cycles required for a significant increase in the reporter’s fluorescence. The data were analyzed with iCycler IQ Real-Time PCR Detection System Software.

Protocol optimization was recommended for developing a good RT-qPCR detection system. According to the manufacturer’s recommendations, the primer was introduced initially at 200 nM in RT-qPCR reactions. The upstream and downstream primers were subjected to an optimization of concentration using a 3 × 3 matrix of 100 nM, 200 nM, and 400 nM for each concentration of primer. This procedure was carried out using RNA resulting from *in vitro* transcription. The optimum concentration was found to be 200 nM for all upstream and downstream primers for the four assays. The concentration of the TaqMan probe was then optimized in order to reduce the quantity used in reactions. Detection of the RBSDV, SRBSDV, UBQ5 and eEF-1a target by RT-qPCR were efficient and reproducible with 400 nM, 200 nM, 200 nM, 200 nM TaqMan probes, respectively. The parameters of the reaction program were examined to determine the most suitable program. Different *Tm* was studied by gradient from 58°C to 63°C. The optimum *Tm* was 10 s at 60°C (the manufacturer’s recommendation was 10 s). The most suitable program and parameter was reverse transcription of the viral RNA at 42°C for 5 min. PCR performed with the hot-start *Taq* polymerase included an enzyme activation step (95°C for 5 s) followed by 40 cycles of denaturation/annealing–extension (10 s at 95°C; 30 s at 60°C).

Viral RNA transcripts, prepared as described above, were used in tenfold serial dilution to generate standard curves and to determine the assay efficiency and the quantification of viral target in the unknown samples.

### Duplex RT-qPCR optimization

In order to avoid interaction, Probe and primer specific assay on different targets were prepared by the single RT-qPCR. To the specific of probe of RBSDV, We used standard sample of RBSDV (10^5^ copies) as template, RBSDV-F/R as primer, SRBSDV-P as probe; to the control, standard sample of RBSDV (10^5^ copies) as template, RBSDV-F/R as primer, RBSDV-P as probe, and then running the program. To the specific of probe of SRBSDV, We used standard sample of SRBSDV (10^5^ copies) as template, SRBSDV-F/R as primer, RBSDV-P as probe; to the control, standard sample of SRBSDV (10^5^ copies) as template, SRBSDV-F/R as primer, SRBSDV-P as probe, and then running the program. The program was that viral RNA Standard sample at 42°C for 5 min. PCR performed with the hot-start *Taq* polymerase included an enzyme activation step (95°C for 5 s) followed by 40 cycles of denaturation/annealing–extension (10 s at 95°C; 30 s at 60°C)

Simultaneously, primer specific assay for different targets was as follows: for the specificity of primer of RBSDV, we used standard sample of RBSDV (10^5^ copies) as template, SRBSDV-F/R as primer, RBSDV-P as probe. As a control, standard sample of RBSDV (10^5^ copies) as template, RBSDV-F/R as primer, RBSDV-P as probe, we run the program; for the specific of primer of SRBSDV, we used standard sample of SRBSDV (10^5^ copies) as template, RBSDV-F/R as primer, SRBSDV-P as probe; as a control, standard sample of SRBSDV (10^5^ copies) as template, SRBSDV-F/R as primer, SRBSDV-P as probe, we run the program. The program for transcription of the viral RNA was at 42°C for 5 min. PCR performed with the hot-start *Taq* polymerase included an enzyme activation step (95°C for 5 s) followed by 40 cycles of denaturation/annealing–extension (10 s at 95°C; 30 s at 60°C). The data were analyzed with iCycler IQ Real-Time PCR Detection System Software. According to the results of single RT-qPCR, the four segments have the same Tm, so we used the 60°C as the Tm value of duplex RT-qPCR. In the duplex reaction, every reaction contained the primers and probes of SRBSDV and RBSDV, we used standard sample of RBSDV (10^5^ copies/μl) and H2O or SRBSDV (10^5^ copies/μl) and H2O as template, respectively. The upstream and downstream primers were subjected to an optimization of concentration using a 3 × 3 matrix of 100 nM, 200 nM, and 400 nM for each concentration of primer. The range of concentration of the TaqMan probe was 100 nM, 200 nM, and 400 nM, respectively. The program for transcription of the viral RNA was at 42°C for 5 min. PCR performed with the hot-start *Taq* polymerase included an enzyme activation step (95°C for 5 s) followed by 40 cycles of denaturation/annealing–extension (10 s at 95°C; 30 s at 60°C). The data were analyzed with iCycler IQ Real-Time PCR Detection System Software.

### Duplex RT-qPCR protocol

We have developed a following protocol for duplex RT-qPCR assay by One Step PrimerScript RT-PCR Kit (Takara, China) as follows: Each 20 μl reaction contained 2 μl of mixed RNA; 10 μl of 2x one Step RT-PCR Buffer III; 0.6 μl of TaKaRa Ex Taq HS; 0.6 μl of PrimeScript RT Enzyme MixII; 0.4 μl (200 nM), 0.4 μl (200 nM), 0.8 μl (400 nM) of SRB-F/R/P and 0.4 μl (200 nM), 0.4 μl (200 nM), 0.4 μl (200 nM) of RB-F/R/P; 3 μl of RNase Free H_2_O. The optimized program was 42°C 5 min; 95°C 10s, 95°C 5 s, 60°C 30 s, going over 40 cycles. Assays were performed on IQ2 real-time PCR system (Bio Rad, USA).

Viral RNA transcripts, prepared as described above, were used in 10-fold serial dilutions to generate standard curves and to determine the assay efficiency.

## Competing interest

The authors declare that they have no competing interests.

## Authors’ contributions

PZ contributed to the design of the study, primer design for the RT-qPCR assay, sample collection, RNA extractions, optimization of the RT-qPCR assay, screening of samples, statistical analysis, designing the duplex RT-qPCR, validation of RT-qPCR assay with duplex RT-qPCR and drafting the manuscript. TTM designed primers for the RT-qPCR assay, contributed to the design of the study, sample collection, RNA extraction and drafting of the manuscript. WL contributed to the design of the study, primer design, sample collection, statistical analysis and designing the duplex RT-qPCR protocol. LL contributed to the design of the study, sample collection and drafting of the manuscript. XW contributed to the design of the study, sample collection, data analysis and drafting the manuscript. All authors read and approved the final manuscript.

## Supplementary Material

Aditional file 1 **Provided detail information of primers and probes of RT-qPCR and RT-PCR used in this experiment.** In the table S-1, Primers and probes for duplex RT-qPCR in this experiment could be expressly seen. Table S-2 provided primers to be used in the process of preparing standard samples. And Primers in the Table S-3 were ready for duplex RT-PCR. Click here for file
