# Supplementary material for: Simultaneous detection and differentiation of Rice black streaked dwarf virus (RBSDV) and Southern rice black streaked dwarf virus (SRBSDV) by duplex real time RT-PCR
Source: Virol J. 2013 Jan 18;10:24. doi: 10.1186/1743-422X-10-24 (PMC3610162; doi:10.1186/1743-422X-10-24)
Supplement: Aditional file 1 — Provided detail information of primers and probes of RT-qPCR and RT-PCR used in this experiment. In the table S-1, Primers and probes for duplex RT-qPCR in this experiment could be expressly seen. Table S-2 provided primers to be used in the process of preparing standard samples. And Primers in the Table S-3 were ready for duplex RT-PCR. [file 1743-422X-10-24-S1.docx]

**List of additional material**

Table S-1: Primers and Probes for duplex qRT-PCR

| Target | Forward and reverse primers (5′–3′) | Start（bp） | Accession number |
| --- | --- | --- | --- |
| RBSDV  SRBSDV  eEF-1a  UBQ5 | F：CATCAAAAAAGCCGGAAGCT  R： CAACCATGATCCCTGTAAGAATAAAA  P： HEX-CGTCACTTTACCATTTGCTTGGCGACA  F：TGAAGTTTCAGAGCACATTCGAA  R：CACCTGGAACTAAAGGCAAAGAA  P：FAM-CGAAAGCCGTTTTCTCAGTCCTTATGCA  F：CCCAAGAGGCCATCAGACA  R：GCCAATACCACCGATCTTGTACA  P：FAM-CCCCTGCGTCTTCCCCTTCAGG  F：AGTGCGGCCTCACCTACGT  R：CCGCCCCCAAAGAACAG  P：FAM-ACCAGCAGGCTTAGGCGTAGGCT | 2420  2560  2453  1460  1545  1490  772  840  793  533  581  557 | AJ409146.1  FN563992.1  AK061464.1  AK061988.1 |

Table S-2: Primers for standard samples of qRT-PCR

| Target | Forward and reverse primers (5′–3′) | Start（bp） | Accession number |
| --- | --- | --- | --- |
| RBSDV  SRBSDV  eEF-1a  UBQ5 | F：GCAAACGCTCGTCATCTAAG  R：CCACCAAACGCTATTTCACT  R-S：CCCGGGCCACCAAACGCTATTTCACT  F：ACGCTGATACCAACAGACCA  R：TTTAGCACCAAGAAAGACGA  R-S：CCCGGGTTTAGCACCAAGAAAGACGA  F：GACAAGATTCCCTTCGTTCC  R：TGTAAATACCCGCATTCCAC  R-S：CCCGGGTGTAAATACCCGCATTCCAC  F：CTCGCCGACTACAACATCC  R：AGGGCATCACAATCTTCACA  R-S：CCCGGG AGGGCATCACAATCTTCACA | 751  1921  1703  2605  652  1596  268  708 | FN563992.1  AJ409146.1  AK061464.1  AK061988.1 |

CCCGGG: Restriction enzyme cutting site of *Sma* I

Table S-3: Primers for duplex RT-PCR

| Target | Forward and reverse primers (5′–3′) | Start（bp） | Accession number |
| --- | --- | --- | --- |
| SRBSDV  RBSDV | F：CGAAACCAGCACTCTACCGAAC  R：AATGAGGAGACTCCGCTCCATG  F：TACTCGCCATGCTTGGTCTA  R：CAACGAAATCAACGCTCACT | 261  881  2291  3260 | EU523360.1  AJ293984.1 |
